# Duration of Treatment Effect Using IncobotulinumtoxinA for Upper-limb Spasticity: A *Post-hoc* Analysis

**DOI:** 10.3389/fneur.2020.615706

**Published:** 2021-01-22

**Authors:** Petr Kaňovský, Elie P. Elovic, Angelika Hanschmann, Irena Pulte, Michael Althaus, Reinhard Hiersemenzel, Christina Marciniak

**Affiliations:** ^1^Faculty of Medicine and Dentistry and University Hospital, Palacký University Olomouc, Olomouc, Czechia; ^2^Moss Rehabilitation, Philadelphia, PA, United States; ^3^Merz Pharmaceuticals GmbH, Frankfurt am Main, Germany; ^4^Department of Physical Medicine and Rehabilitation and the Department of Neurology, Northwestern University Feinberg School of Medicine and Shirley Ryan AbilityLab, Chicago, IL, United States

**Keywords:** duration of effect, incobotulinumtoxinA, post-stroke, upper-limb spasticity, treatment interval

## Abstract

The efficacy and safety of incobotulinumtoxinA ≤400 U was demonstrated in subjects with post-stroke upper-limb spasticity in a randomized, double-blind Phase 3 study with an open-label extension (OLEX; EudraCT number 2005-003951-11, NCT00432666). We report a *post-hoc* analysis of the duration of the treatment effect. Subjects completing the placebo-controlled main period (single injection cycle with 12–20-week observation) entered the OLEX and received a maximum of five further treatments (maximum duration 69 weeks) with incobotulinumtoxinA ≤400 U at flexible intervals with a minimum duration of 12 weeks, based on clinical need. Intervals between two consecutive incobotulinumtoxinA injections, excluding treatment intervals prior to the end-of-study visit, were evaluated. Of 437 incobotulinumtoxinA treatment intervals, 415 received by 136 subjects were included in the *post-hoc* analysis. More than half (52.3%; 217/415) of all incobotulinumtoxinA reinjections were administered at Week ≥14, 31.1% (129/415) at Week ≥16, 19.0% (79/415) at Week ≥18, and 11.6% (48/415) at Week ≥20. The duration of effect may vary and can exceed 20 weeks or more, which was observed in at least one injection cycle in 29.4% (40/136) subjects over the course of their treatment. Data show that incobotulinumtoxinA retreatment for upper-limb spasticity may not be required at 12-week intervals and provides evidence for flexible treatment intervals beyond this time frame.

## Introduction

Spasticity is a key feature of functional impairment following a stroke, with prevalence in stroke survivors increasing over time and affecting up to 42.6% of stroke survivors in the chronic phase 3–6 months post-stroke (1, 2). Post-stroke spasticity can have detrimental effects on an individual's ability to perform activities of daily living (1, 3, 4), leading to an increased burden on caregivers (3) and directly affecting quality of life (1, 3–6). Therefore, the principal goals in the management of spasticity are to improve function and quality of life, and prevent further impairments in affected limbs, while providing symptomatic relief (7).

Botulinum neurotoxin type A (BoNT-A) injections are recommended as an effective treatment option for spasticity of the upper limb (8–10). IncobotulinumtoxinA (Xeomin®, Merz Pharmaceuticals GmbH, Frankfurt am Main, Germany) is a BoNT-A free from complexing proteins, which is approved for the treatment of upper-limb spasticity at doses up to 400 units (U) in the USA and 500 U in Europe, with dosing intervals ≥12 weeks (11, 12). The efficacy and safety of incobotulinumtoxinA in subjects with upper-limb spasticity have been demonstrated in several clinical trials (13–18). Where repeat injections were administered, subjects received reinjections at fixed 12-week (13, 14) or 12–16-week intervals (18). The design of this Phase 3, randomized, double-blind, placebo-controlled, multicenter trial with open-label extension (OLEX, EudraCT number 2005-003951-11, NCT00432666) enabled subjects to receive a maximum of six injections with a flexible dose (≤400 U, with mean doses between 307 U and 363 U per cycle,) and dosing interval (≥12 weeks) based on clinical need, as agreed by the subject and investigator (15, 16). The flexible dosing paradigm employed in this study is of relevance to real-world clinical practice. An earlier survey of subjects with post-stroke spasticity treated with BoNT-A showed that the mean (standard deviation, SD) treatment interval was 13.7 (3.5) weeks (19). Satisfaction with BoNT treatment was high overall, and at its lowest prior to reinjection, suggesting a need for individualized treatment. Furthermore, when questioned about treatment intervals, a high proportion of subjects said that they would prefer flexible treatment intervals (19). In subjects with cervical dystonia (20) and blepharospasm (21), repeated incobotulinumtoxinA treatment at flexible intervals of 6–20 weeks based on individual treatment requirement resulted in sustained efficacy with no new or unexpected safety concerns or significant differences in the incidence of adverse events at different treatment intervals. Here, we report the results of a *post-hoc* analysis of the duration of the incobotulinumtoxinA treatment effect in the study detailed above (15, 16), using treatment interval data across up to five complete incobotulinumtoxinA injection cycles for upper-limb spasticity.

## Materials and Methods

### Study Design and Subjects

Details of the study design and subject eligibility criteria have been reported previously (15, 16). Briefly, the key inclusion criteria included a history of stroke ≥6 months prior to enrolment leading to focal spasticity of wrist and finger flexors (presence of the respective clinical patterns and Ashworth Scale [AS] score ≥2), and a Disability Assessment Scale score ≥2 in 1 of 4 domains (dressing, limb position, pain, and hygiene) selected as the principal therapeutic target. All subjects who completed the double-blind, placebo-controlled main period (MP, a single set of incobotulinumtoxinA or placebo injections with an assessment period of 12–20 weeks) could enter the OLEX, where they received incobotulinumtoxinA at total doses of ≤400 U at flexible intervals ≥12 weeks. Subjects could be BoNT-naïve, or could have received prior BoNT treatment for spasticity. Any antispastic medication, physical and occupational therapy, or other rehabilitation treatment had to be stable for at least 2 weeks prior to screening and throughout the MP. Upper-limb physical or occupational therapy was not permitted on study visit days to prevent any impact on evaluations of spasticity.

If there was no need for reinjection at the MP Week 12 visit, subjects were followed-up by telephone contact or optional scheduled visits every 2 weeks until Week 20 post-treatment. If no new injection was required at Week 20, the subject was not removed from the study, but entered the OLEX period without reinjection. During the OLEX, the study design allowed for full flexibility in the choice of the time for reinjection after Week 12 of each cycle, and there was no artificially fixed schedule requiring treatment only at specific visits. Subjects returned for assessment 4 weeks post-treatment and were retreated as required when treatment effects waned. Subjects were contacted once by telephone if they did not present for reinjection within 20 weeks; the latest timepoint for reinjection was Week 48.

Repeat injections were administered if the subject expressed a need for reinjection, and the investigator agreed on this need. If subjects expressed a need for reinjection, a visit had to be scheduled immediately (i.e., within the following 7 days). The investigator's decision to reinject was based on AS scores in all treated muscle groups reaching study or cycle baseline levels (compulsory injection), or based on clinical experience in all other cases (e.g., improvement in AS scores in some treated muscle groups). If there was no investigator-determined clinical need for retreatment, subjects were asked to return for reassessment within 4 weeks and the visit was documented as an optional visit. The number of optional visits therefore indicates diverging assessment of the need for retreatment by the investigator and subject resulting in postponement of the subject's treatment. Since the treatment interval duration was based on clinical need for retreatment, it also indicated the duration of treatment effect. The maximum duration of the OLEX was 69 weeks, with a maximum of five repeat injections for each subject. The number of injections possible within the overall study time frame was thus reduced in subjects with a longer duration between consecutive injections.

### Statistical Analyses

Intervals between two consecutive incobotulinumtoxinA injections in the MP and OLEX were evaluated. Treatment intervals prior to the end-of-study visit that may have been influenced by the maximum allowed study duration, irrespective of clinical need, were excluded.

To account for outliers and factors other than a clinical need for reinjection (e.g., visit scheduling), duration thresholds were applied, and the number of subjects with at least one treatment above the threshold(s) (Week ≥14, 16, 18, or 20) was calculated.

Treatment intervals between a placebo injection in the MP and the first incobotulinumtoxinA injection in the OLEX were evaluated for comparison. The difference in treatment interval duration between all incobotulinumtoxinA injections vs. the interval between the MP placebo injection and the first incobotulinumtoxinA injection in the OLEX was assessed using a *t*-test.

## Results

### Subjects

**Figure 1:**
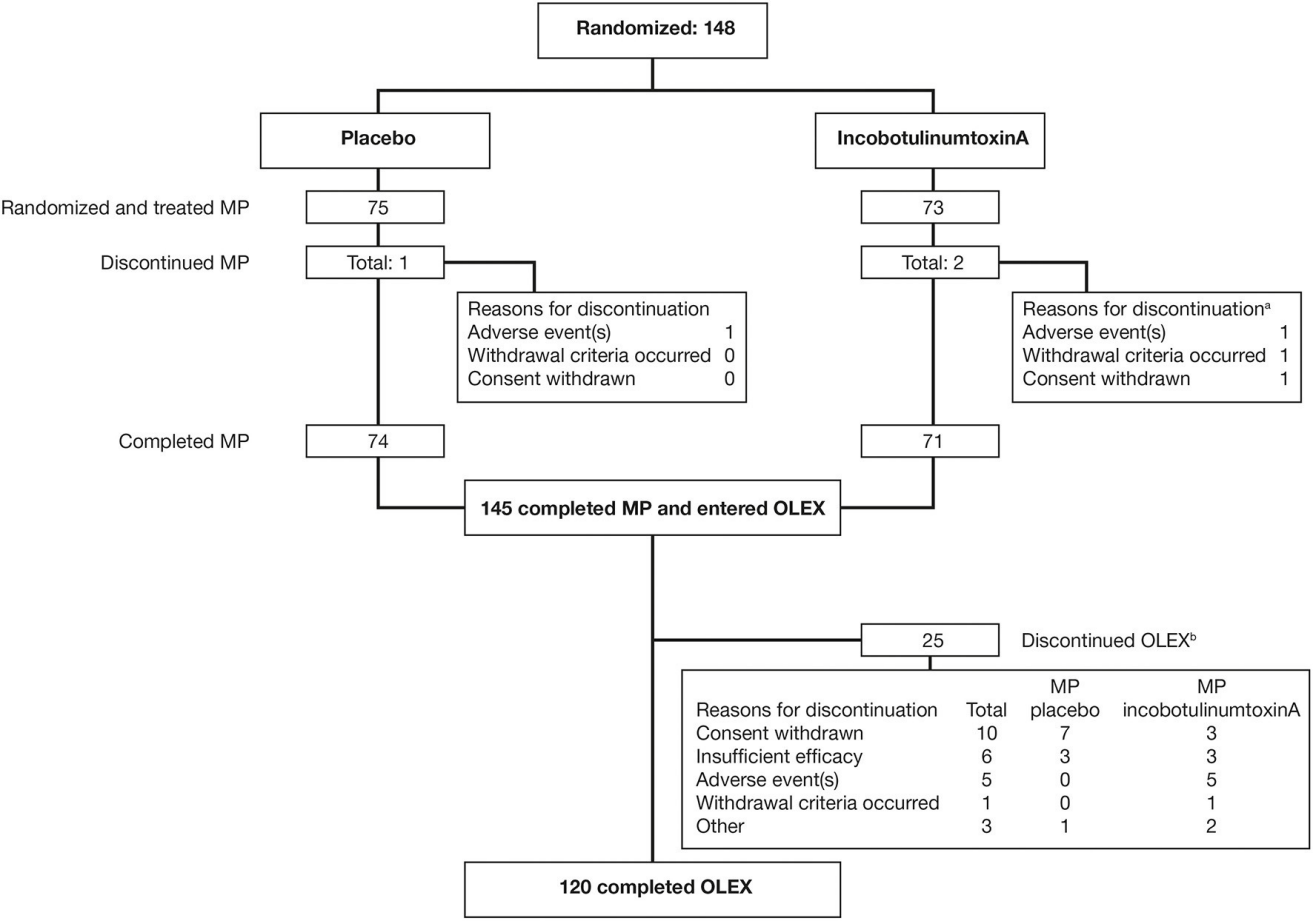
Subject flow through the MP and OLEX. ^a^Multiple reasons for discontinuation were possible; ^b^Main reason for discontinuation; multiple entries were possible. MP, main period; OLEX, open-label extension.

Of the 148 subjects randomized, 145 (98.0%) completed the MP and entered the OLEX, and 120 (81.1%) completed the OLEX (Figure 1). The baseline characteristics of the OLEX population have been described previously (16); briefly, 64.1% of subjects were male, the mean (SD) age was 55.7 (12.1) years and time since the first diagnosis of spasticity at the MP baseline was 55.0 (48.7) months, and 36 (24.3%) had been previously treated with BoNT for upper-limb spasticity. One subject had no incobotulinumtoxinA injections and was excluded from this *post-hoc* analysis.

### Treatment Interval Duration (Duration of Treatment Effect)

**Figure 2:**
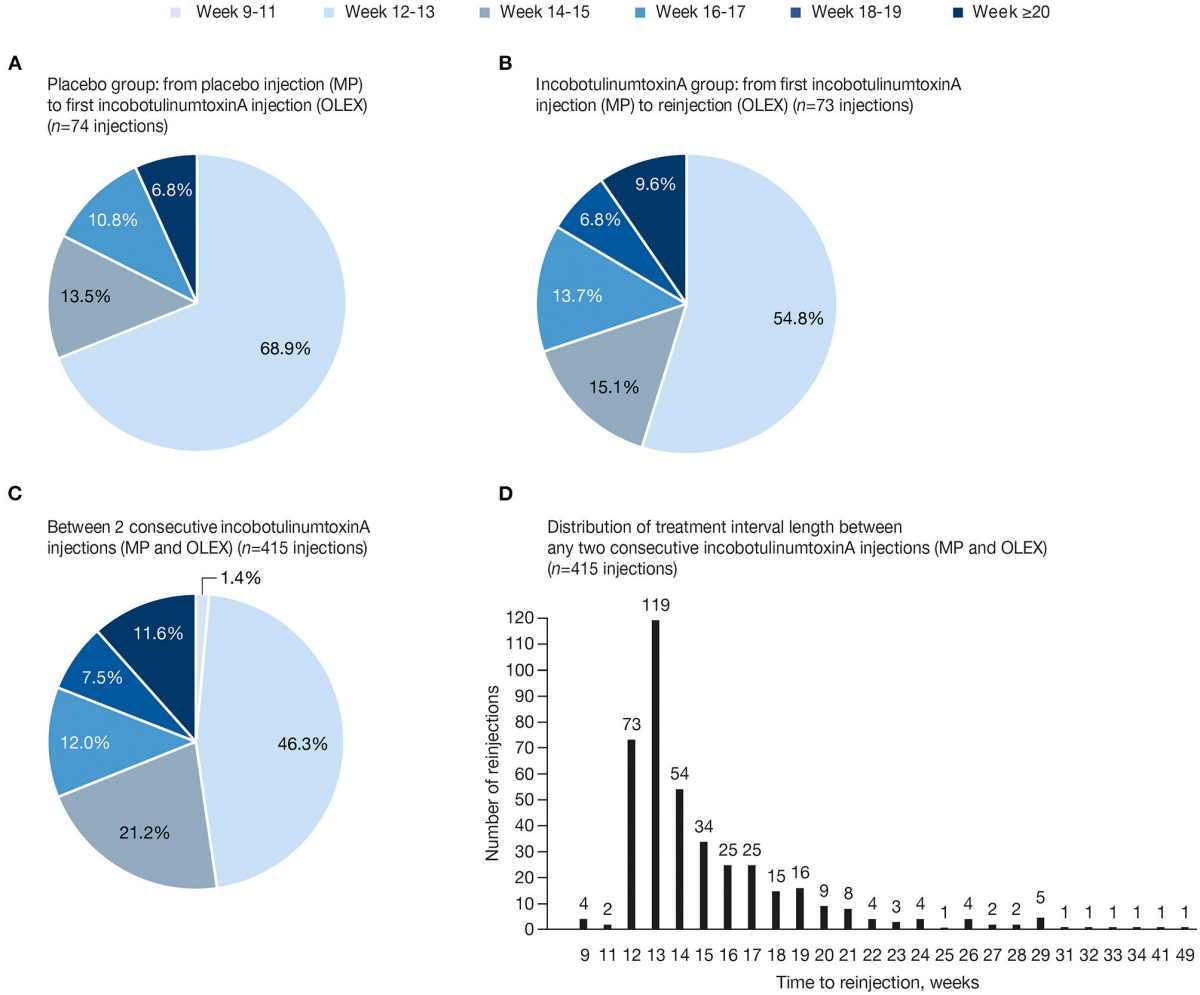
Percentage of reinjections between treatments in each time frame **(A)** MP placebo to first incobotulinumtoxinA injection in the OLEX; **(B)** MP incobotulinumtoxinA to first incobotulinumtoxinA reinjection in the OLEX; **(C)** any two consecutive incobotulinumtoxinA injections, including the initial MP injection and reinjections in the OLEX; and **(D)** distribution of treatment interval length until reinjection, in weeks, between any two consecutive incobotulinumtoxinA injections, including MP and OLEX injections. MP, main period; OLEX, open-label extension.

Of 437 incobotulinumtoxinA treatment intervals, 415 from 136 subjects, excluding intervals prior to the end-of-study visit, were included in the *post-hoc* analysis. The earliest reinjection was at Week 9 and the latest at Week 49 (Figure 2). A total of 74 intervals between a MP placebo injection and the first incobotulinumtoxinA injection in the OLEX were included for comparison. Optional visits indicating diverging assessment by the investigator and subject on the need for reinjection, resulting in postponement of reinjection, were recorded for 23/415 (5.5%) incobotulinumtoxinA treatment intervals, while this was recorded for 6/74 (8.1%) intervals between MP placebo and incobotulinumtoxinA. The mean (SD) interval between the MP injection and the first incobotulinumtoxinA injection in the OLEX was 14.0 (2.19) weeks in the placebo group (median [range] 13.0 [12–21] weeks) vs. 14.8 (2.76) weeks in those who received incobotulinumtoxinA (median [range] 13.0 [12–23] weeks; *p* < 0.05, *t*-test vs. MP placebo cycle interval). Across all consecutive incobotulinumtoxinA injections, the mean (SD) treatment interval was 15.3 (4.48) weeks (median [range] 14.0 [9.0–49.0] weeks).

Between a placebo injection in the MP and the first incobotulinumtoxinA treatment in the OLEX, 31.1% of intervals were at Week ≥14, 17.6% at Week ≥16, and 6.8% at Week ≥18 (Figure 2A). Between an incobotulinumtoxinA injection in the MP and the first incobotulinumtoxinA reinjection in the OLEX, 45.2% of intervals were at Week ≥14, 30.1% at Week ≥16, 16.4% at Week ≥18, and 9.6% at Week ≥20 (Figure 2B). When all intervals between incobotulinumtoxinA injections in all injection cycles were taken into account, more than half (52.3%) of incobotulinumtoxinA reinjections were administered at Week ≥14, 31.1% at Week ≥16, 19.0% at Week ≥18, and 11.6% at Week ≥20 (Figure 2C). Most subjects received reinjections at intervals ≤ 28 weeks (409/415 intervals, 98.6%); 5 (1.2%) treatments were administered at Week 29 (effect duration of 28 weeks) and 6 (1.4%) administered at Week ≥29 were in single cases (Figure 2D).

**Figure 3:**
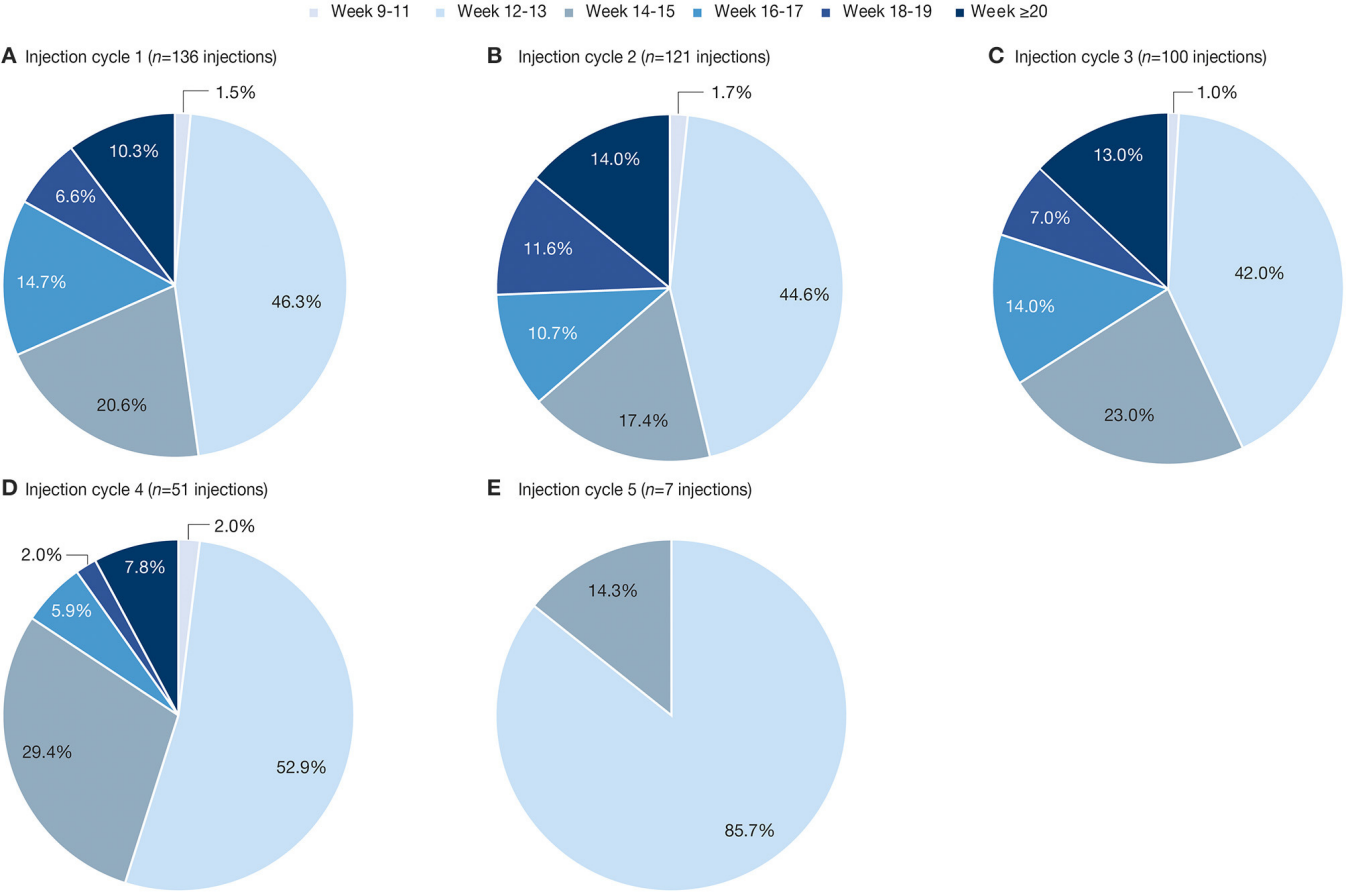
Percentage of reinjections at each timepoint between any two consecutive incobotulinumtoxinA injections in the OLEX **(A)** injection cycle 1; **(B)** injection cycle 2; **(C)** injection cycle 3; **(D)** injection cycle 4; **(E)** injection cycle 5. OLEX, open-label extension.

The distribution and maximum duration of treatment intervals did not change with an increasing number of injection cycles, except for the fifth and last cycle of this analysis (Figure 3). Shorter treatment intervals in this cycle are due to the upper limit of the overall observation period being 69 weeks. Of note, the mean [SD] total incobotulinumtoxinA doses administered were comparable across all treatment intervals of the OLEX period (ranging from 339.4 [87.8] to 363.1 [67.9] U).

### Distribution of Treatment Interval Duration Per Subject

**Figure 4:**
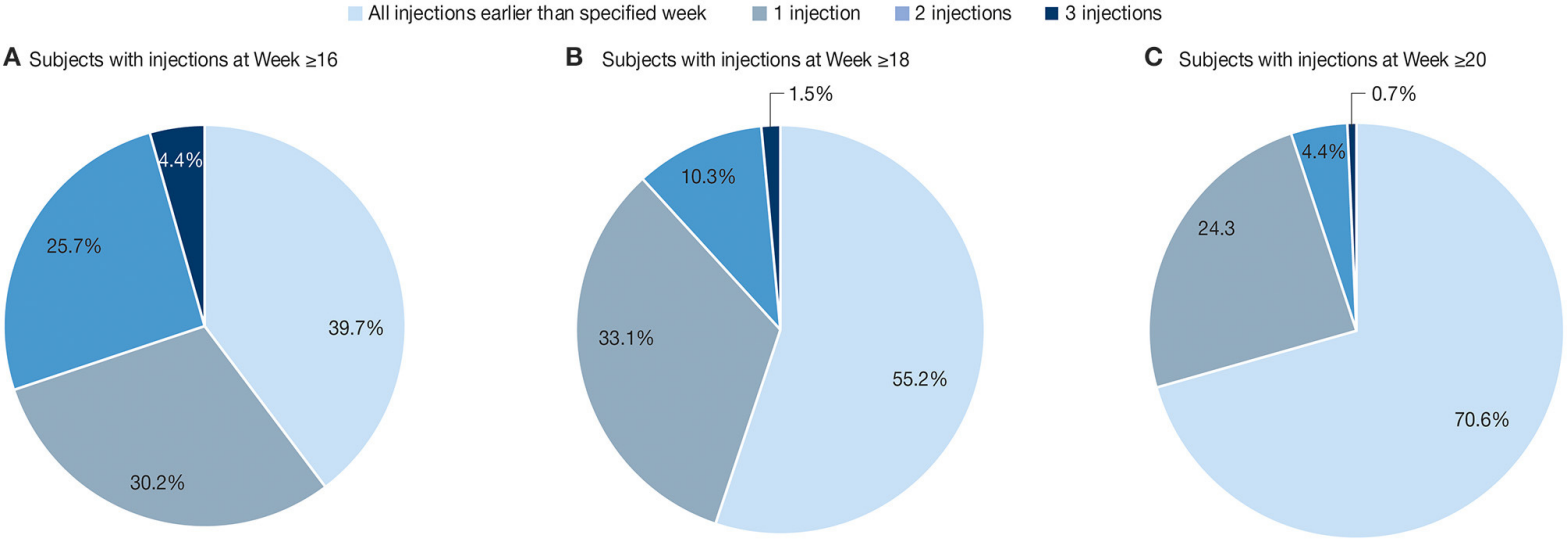
Percentage of subjects (*N* = 136) with 0 (none), 1, 2, or 3 reinjections at **(A)** Week ≥16; **(B)** Week ≥18; **(C)** Week ≥20. Frequency of treatment intervals. The results are descriptive. All treatment intervals meeting the duration thresholds were counted, and subjects could be counted in more than one category.

While the above analyses were based on the total number of injection cycles, we also investigated the distribution of treatment interval durations per subject. Among the 136 subjects with treatment intervals between incobotulinumtoxinA injections, 82 (60.3%) had one or more treatments at Week 16 or later. Of these, 41 subjects (30.1%) had one, 35 (25.7%) had two, and 6 (4.4%) had three treatments at Week ≥16. Furthermore, 61 (44.9%) and 40 (29.4%) of 136 subjects analyzed had at least one treatment at Week ≥18 and Week ≥20, respectively, over the course of their treatment (Figure 4).

## Discussion

This *post-hoc* analysis of treatment interval duration is the first to show detailed data on the duration of incobotulinumtoxinA treatment effect over up to five complete injection cycles. Results demonstrate a duration of treatment effect beyond 12 weeks with incobotulinumtoxinA in subjects with post-stroke upper-limb spasticity, with 31.1% of all reinjections performed at 16 weeks or longer following the previous treatment and 60.3% of subjects having at least one injection interval of 16 weeks or longer. These results are consistent with those presented in a similar analysis of the duration of treatment effect in four clinical studies with abobotulinumtoxinA (22), which included data from an OLEX study of abobotulinumtoxinA ≤1,500 U at intervals ≥12 weeks for the treatment of upper-limb spasticity (23). During this OLEX study, 34.9% of subjects received a second injection at Week ≥16, and 24.0% received a third injection at Week ≥16 (22, 23). However, while abobotulinumtoxinA reinjections were possible only at fixed visits every 4 weeks from Week 12 to Week 24 post-treatment (22, 23), the design of the current incobotulinumtoxinA study allowed for full flexibility in the timing of the visits, thereby avoiding artificial overestimation of the duration of treatment effect. It has been hypothesized that a long duration of treatment effect is related to greater amounts of active neurotoxin injected in approved doses of abobotulinumtoxinA compared with approved doses of other BoNT-A formulations (22). However, this hypothesis is not confirmed by the results of the current study in which similar duration of treatment effect was observed with incobotulinumtoxinA.

Several patient and practitioner surveys of the use of BoNT-A in spasticity and cervical dystonia have highlighted a desire for individualized treatment intervals (19, 24, 25). In the present study, the lack of a requirement for the subjects to return at a predefined interval for reassessment and reinjections likely more closely represents injection intervals based on their clinical need. Furthermore, the low rate of documented optional visits supports agreement between the investigator and the subject that reinjection was needed at the time-point reported for the reinjection. In contrast to surveys or retrospective chart analyses, this Phase 3 study design largely controlled for major confounders such as dose, concomitant therapies, and economic divergences, and the similar distribution of interval lengths with incobotulinumtoxinA treatment under double-blind conditions in the MP (Figure 2B) and the following open-label cycles (Figure 3) suggests that no specific bias was introduced by the open-label design. Furthermore, with repeated treatment during the OLEX, the majority of adverse events (AEs) occurred in the first two OLEX injection cycles (16). In addition, the incidence of AEs considered to be related to treatment by the investigator, which may have led to postponement of treatment, was low in the overall study population, occurring in 5/148 (3.4%) subjects (2 incobotulinumtoxinA and 3 placebo recipients) in the MP (15) and 16/145 (11.0%) subjects over all injection cycles in the OLEX, including muscle weakness in 5/145 (3.4%) subjects (16). Therefore, based on the information from up to six injection cycles, the occurrence of treatment-related AEs would not be expected to have influenced the overall length of treatment intervals.

The results of the current *post-hoc* analysis showed individual variability in the duration of incobotulinumtoxinA treatment effect based on the intervals between injections. The mean (SD) treatment interval between the MP injection and the first incobotulinumtoxinA injection in the OLEX was 14.0 (2.19) weeks in the placebo group and 14.8 (2.76) weeks in the incobotulinumtoxinA group, which is similar to the 13.7 (3.5) weeks previously reported in a survey of subjects with post-stroke spasticity receiving BoNT-A treatment (19). Although statistically significant, differences between groups were relatively small in the MP of the current study, which may reflect a placebo response consistent with perceived efficacy of the initial treatment. In the authors experience in clinical practice, patients' perceived effects of the first injection are often greater than at subsequent injections where they may return for a repeat injection around 12 weeks post-treatment although the effects have not fully worn off and nascent EMG readings suggest continuing motor unit remodeling (26). In the current study, taking into account only incobotulinumtoxinA treatment intervals, a treatment effect of ≥20 weeks was observed in almost one-quarter of subjects in at least one injection cycle, with most reinjections occurring ≤Week 29, while treatments beyond Week 29 occurred in a few outlier subjects and could be more susceptible to confounding factors. Therefore, with incobotulinumtoxinA doses of ≤400 U, 28 weeks may be considered as the longest duration of effect achievable in some subjects. In clinical practice, entirely flexible and individualized reinjection schedules may encounter organizational limits. However, these results could help clinicians feel more comfortable to offer treatment at intervals based on patients' needs. Assessing the ongoing effect of the previous injection could allow subsequent injections to be arranged with longer treatment intervals. Greater duration of effect, and thus longer treatment intervals, may also alleviate the burden on subjects and caregivers associated with frequent reinjection.

A strength of this analysis is the long observation period allowing for a flexible number of injection cycles triggered by subjects' needs. Limitations of our research are the *post-hoc* character of the statistical analyses and the fact that we could not control for every confounding factor that may have influenced the timing of reinjection, e.g., time conflicts, organizational reasons, or acute illnesses.

## Conclusions

Together with the efficacy and safety data reported previously (15, 16), these data support the use of flexible treatment intervals beyond 12 weeks tailored to individual clinical need in the management of post-stroke upper-limb spasticity, with longer intervals ≥20 weeks possible in some subjects receiving repeated incobotulinumtoxinA treatment.

## Data Availability Statement

The datasets for this study are available on request to the corresponding author.

## Ethics Statement

The studies involving human participants were reviewed and approved by the local ethics committees at each participating site. Subjects provided written informed consent prior to partaking in any study procedures. The study was registered in the European Union Clinical Trials Register (EudraCT number 2005-003951-11) and the United States National Library of Medicine (clinicaltrials.gov study ID NCT00432666), and conducted in accordance with Good Clinical Practice and the Declaration of Helsinki.

## Author Contributions

PK, EPE, MA, and IP contributed to study conceptualization and design. PK, EPE, and CM contributed to the investigation. AH and MA performed formal analysis of the data. MA wrote the original draft manuscript. All authors contributed to the revision and critical review of the manuscript and approved the final version for publication.

## Conflict of Interest

PK has received honoraria and speakers fee from Desitin/Merz Pharmaceuticals, Medtronic, EverPharma, and AbbVie. EPE participated in a Speakers' Bureau for Ipsen Biopharmaceuticals Inc. and has received funding from Merz Pharmaceuticals to participate as coordinating investigator for study 3001. AH, IP, MA, and RH are employees of Merz Pharmaceuticals GmbH. CM is employed by Shirley Ryan AbilityLab, formerly known as the Rehabilitation Institute of Chicago. This institution received funding from Merz Pharmaceuticals for work performed for study 3001. The Shirley Ryan AbilityLab also receives funding from Ipsen Biopharmaceuticals Inc. and Revance Therapeutics Inc. for CM's research-related activities.
